# Adult-specific Reelin expression alters striatal neuronal organization: implications for neuropsychiatric disorders

**DOI:** 10.3389/fncel.2023.1143319

**Published:** 2023-04-20

**Authors:** Mònica Pardo, Sara Gregorio, Enrica Montalban, Lluís Pujadas, Alba Elias-Tersa, Núria Masachs, Alba Vílchez-Acosta, Annabelle Parent, Carme Auladell, Jean-Antoine Girault, Miquel Vila, Angus C. Nairn, Yasmina Manso, Eduardo Soriano

**Affiliations:** ^1^Developmental Neurobiology and Regeneration Laboratory, Department of Cell Biology, Physiology and Immunology, Institute of Neurosciences, Universitat de Barcelona, Barcelona, Spain; ^2^Centro de Investigación Biomédica en Red Enfermedades Neurodegenerativas (CIBERNED), Instituto de Salud Carlos III, Madrid, Spain; ^3^Institut du Fer à Moulin UMR-S 1270, INSERM, Sorbonne University, Paris, France; ^4^Department of Experimental Sciences and Methodology, Faculty of Health Science and Welfare, University of Vic – Central University of Catalonia (UVic-UCC), Vic, Spain; ^5^Tissue Repair and Regeneration Laboratory (TR2Lab), Institut de Recerca i Innovació en Ciències de la Vida i de la Salut a la Catalunya Central (IRIS-CC), Barcelona, Spain; ^6^Neurodegenerative Diseases Research Group, Vall d’Hebron Research Institute, Barcelona, Spain; ^7^Department of Biochemistry and Molecular Biology, Autonomous University of Barcelona (UAB), Barcelona, Spain; ^8^Institució Catalana de Recerca i Estudis Avançats (ICREA), Barcelona, Spain; ^9^Aligning Science Across Parkinson’s (ASAP) Collaborative Research Network, Chevy Chase, MD, United States; ^10^Department of Psychiatry, Yale University School of Medicine, New Haven, CT, United States

**Keywords:** Reelin, striatum, interneurons, dopamine projections, schizophrenia, Tourette’s syndrome

## Abstract

In addition to neuronal migration, brain development, and adult plasticity, the extracellular matrix protein Reelin has been extensively implicated in human psychiatric disorders such as schizophrenia, bipolar disorder, and autism spectrum disorder. Moreover, heterozygous *reeler* mice exhibit features reminiscent of these disorders, while overexpression of Reelin protects against its manifestation. However, how Reelin influences the structure and circuits of the striatal complex, a key region for the above-mentioned disorders, is far from being understood, especially when altered Reelin expression levels are found at adult stages. In the present study, we took advantage of complementary conditional gain- and loss-of-function mouse models to investigate how Reelin levels may modify adult brain striatal structure and neuronal composition. Using immunohistochemical techniques, we determined that Reelin does not seem to influence the striatal patch and matrix organization (studied by μ-opioid receptor immunohistochemistry) nor the density of medium spiny neurons (MSNs, studied with DARPP-32). We show that overexpression of Reelin leads to increased numbers of striatal parvalbumin- and cholinergic-interneurons, and to a slight increase in tyrosine hydroxylase-positive projections. We conclude that increased Reelin levels might modulate the numbers of striatal interneurons and the density of the nigrostriatal dopaminergic projections, suggesting that these changes may be involved in the protection of Reelin against neuropsychiatric disorders.

## 1. Introduction

Reelin is an extracellular matrix protein important for neuronal migration and layer formation during neocortical development (D’Arcangelo et al., 1995; Alcántara et al., 1998; Rice and Curran, 2001; Soriano and Del Río, 2005; Cooper, 2008; Hirota and Nakajima, 2017; Vílchez-Acosta et al., 2022). Besides its role during development, the Reelin pathway is also active in the adult brain, controlling glutamatergic neurotransmission, dendritic spine formation, synaptic plasticity and adult neurogenesis (Chen et al., 2005; Herz and Chen, 2006; Qiu et al., 2006b; Groc et al., 2007; Niu et al., 2008; Pujadas et al., 2010; Teixeira et al., 2012; Bosch et al., 2016). Reelin binds to apolipoprotein E receptor 2 (ApoER2) and very-low-density lipoprotein receptor (VLDLR), leading to the phosphorylation and activation of the intracellular adaptor protein Disabled 1 (Dab1), which triggers a complex signaling cascade involving members of the Src kinase family, PI3K, Erk1/2 and GSK3 kinases, and Cullin-5-dependent degradation, amongst others (Howell et al., 1997, 1999; D’Arcangelo et al., 1999; Hiesberger et al., 1999; Beffert et al., 2002; Arnaud et al., 2003; Benhayon et al., 2003; Ballif et al., 2004; Strasser et al., 2004; González-Billault et al., 2005; Simó et al., 2007, 2010; Yasui et al., 2010; Molnár et al., 2019).

Genetic studies have associated the Reelin gene (*RELN*) with a number of psychiatric diseases, including schizophrenia, bipolar disorder, and autism spectrum disorder (Impagnatiello et al., 1998; Fatemi et al., 2001, 2005; Persico et al., 2001; Grayson et al., 2005; Ovadia and Shifman, 2011; Wang et al., 2014; Baek et al., 2015; Lammert and Howell, 2016). This link is also supported by studies showing that Reelin levels are reduced in patients with schizophrenia and bipolar disorder (Fatemi et al., 2000; Torrey et al., 2005; Ruzicka et al., 2007), and can be altered by psychotropic medication (Fatemi et al., 2009). In fact, Reelin haploinsufficiency models, based on the suppression or reduction of Reelin expression (or its downstream pathway), manifest features related to neuropsychiatric disorders, such as cognitive impairments, psychosis vulnerability, and learning deficits that frequently coexist with evident alterations in hippocampal plasticity (Tueting et al., 1999; Krueger et al., 2006; Marrone et al., 2006; Qiu et al., 2006a; Ammassari-Teule et al., 2009; Folsom and Fatemi, 2013). Conversely, overexpression of Reelin protects against psychiatric disease-related phenotypes in mice, since it reduces cocaine sensitization, disruption of pre-pulse inhibition (PPI) and the time spent floating in the forced swim test (Teixeira et al., 2011). Furthermore, Reelin also regulates adult neurogenesis and synaptogenesis (Kim et al., 2002; Pujadas et al., 2010; Teixeira et al., 2012; Bosch et al., 2016), whose disruption is considered to be involved in the pathogenesis of psychiatric disorders (Kempermann, 2008; Zhao et al., 2008).

The striatum plays a critical function in motor control and regulation of motivated behaviors (Bolam et al., 2000). Its neuronal population is composed of 5–10% interneurons but the large majority (90–95%) are efferent neurons, the GABAergic medium spiny neurons (MSNs). The latter can be classified into striatonigral or striatopallidal subtypes based on their axonal projections to the internal globus pallidus (iGP) and substantia nigra (SN) or to the external globus pallidus (eGP), respectively. They can be distinguished by the expression of the dopamine D1 receptor (striatonigral MSNs) or the dopamine D2 receptor (striatopallidal MSNs) (Bolam, 1984; Schiffmann et al., 1991; Gerfen, 1992; Smith et al., 1998). Although the striatum exhibits a relatively uniform appearance, it presents a complex organization based in two different compartments: the patches or striosomes (stained by μ-opioid receptor MOR) and the matrix, which surrounds the patches (Olson et al., 1972; Graybiel and Ragsdale, 1978; Herkenham and Pert, 1981). A proper cellular and compartmental organization is essential for a correct striatal function (Crittenden and Graybiel, 2011).

Despite the involvement of the striatum (including the nucleus accumbens) and its circuitry in psychiatric disorders such as major depression, schizophrenia, and obsessive-compulsive disorder, few studies addressing how Reelin influences striatal structure and circuits are available (de Guglielmo et al., 2022). Most of these studies use heterozygous *reeler* mice as a model, which have reduced Reelin expression also during development. Here we investigate how altering Reelin levels, specifically at late postnatal and adult stages, may lead to cellular and compartmental changes in the striatum that could be related to neuropsychiatric disorders. We used gain- and loss-of-function conditional mouse models to investigate how Reelin levels may modify striatal structure and neuronal composition. Our results suggest that whereas Reelin does not seem to influence the patch-matrix striatal organization and the numbers of MSNs, overexpression of Reelin leads to increased numbers of striatal interneurons and to a slight increase in the dopaminergic projections.

## 2. Materials and methods

### 2.1. Animals

The TgRln is a conditionally regulated transgenic line that overexpresses Reelin from postnatal (P) day 5–10 by means of a transactivator (tTA) under the control of the calcium–calmodulin-dependent kinase II α promoter (pCaMKIIα) (Pujadas et al., 2010). Reelin transgenic littermates, which have an inactive form of the Reelin gene insertion without the transactivator tTA, were used as controls. For the generation of the Reelin conditional knockout mouse line, homozygous floxed Reelin (fR/fR) mice, with the exon 1 of the Reln gene flanked by loxP sites, were crossed with a heterozygous UbiCreERT2 line [B6.Cg-Tg(UBC-cre/ESR1)1Ejb/J, stock #008085, The Jackson Laboratory], both on a C57BL/6J background (Vílchez-Acosta et al., 2022). The UbiCreERT2 line displays a ubiquitous expression of the Cre recombinase fused to a modified estrogen receptor ligand-binding domain that retains the Cre at the cytoplasm. Administration of an estrogen receptor antagonist (tamoxifen) at P45–60 induces the nuclear translocation of Cre recombinase and the ubiquitous scission of the floxed gene sequence (*Reln*) in all tissues at adult stages. The resultant offspring (Cre fR/fR) was used for the experiments, and fR/fR littermates were used as controls. In both transgenic lines, 4–5 months old female and male mice were used for the experiments.

Male, 8–10-week old, Drd2-EGFP (*n* = 20 Swiss-Webster and 6 C57BL/6N background, founder S118), Drd1a-EGFP (*n* = 4 Swiss-Webster and *n* = 4 C57BL/6N background, founder X60) hemizygous mice were also used in this study. BAC Drd2- and Drd1a-EGFP mice, that express the reporter protein enhanced green fluorescent protein under the control of the D2 and D1 receptor promoters, were generated by GENSAT (Gene Expression Nervous System Atlas) at the Rockefeller University (New York, NY, USA) (Gong et al., 2007).

Mice were bred, studied, and processed at the animal research facility of the Faculty of Pharmacy of the University of Barcelona and at the animal research facility of the Rockefeller University. Animals were provided with food and water *ad libitum* and maintained in a temperature-controlled environment in a 12/12 h light-dark cycle. All the experiments involving animals were performed in accordance with the European Community Council directive 2010/63/EU, the National Institute of Health guidelines for the care and use of laboratory animals, and the Rockefeller University’s Institutional Animal Care and Use Committee (protocol 14753-H). Experiments were also approved by the local ethical committees.

### 2.2. PCR genotyping

DNA was extracted from tail biopsies by adding 100 μl sodium hydroxide (50 mM), and incubating at 100°C during 15 min. Then, samples were kept on ice for 10 min and stored at −20°C until use.

The PCR was performed with the GoTaq^®^ Green Master Mix (Promega), and the primers used for genotyping were as follows. Cre fR/fR line: for homozygous floxed Reelin detection, FloxA (5′CGAGGTGCTCATTTCCCTGCACATTGC3′) and FloxB (5′ CACCGACCAAAGTGCTCCAATCTGTCG 3′) primers were used. Homozygous fR/fR mice present only one band of 613 bp whereas heterozygous mice present an additional band at 496 bp. To determine the presence of UbiCre, the primers UbiCre1(5′ GCG GTC TGG CAG TAA AAA CTA TC 3′) and UbiCre2 (5′ GTC AAA CAG CAT TGC TGT CAC TT 3′) which are specific for UbiCreERT2, and UbiCre3 (5′ CTA GGC CAC AGA ATT GAA AGA TCT 3′) and UbiCre4 (5′ GTA GGT GGA AAT TCT AGC ATC ATC C 3′) as internal positive control were used. Mice heterozygous for Cre (Cre fR/fR) had a double band at 324 and 100 bp while mice negative for Cre only amplified the 100 bp band. TgRln line: the primers RLTG-gen-F (5′-TTGTACCAGGTTCCGCTGGT-3′) and RLTG-gen-R (5′-GCA CAT ATC CAG GTT TCA GG-3′) were used to amplify both the endogenous Reelin gene (720 bp) and the transgenic DNA (320 bp); the primers nTTA-C (5′-ACT AAG TCA TCG CGA TGG AG-3′) and nTTA-F (5′-CGA AAT CGT CTA GCG CGT C-3′), were used to detect the transactivator tTA transgene (Pujadas et al., 2010).

### 2.3. Tamoxifen administration

Inactivation of Reelin expression was induced at postnatal day 45–60 by daily intraperitoneal injections of tamoxifen dissolved in 10% alcohol-90% sunflower oil for 3 consecutive days (180 mg/kg/day; Sigma-Aldrich).

### 2.4. Immunohistochemistry

For immunohistochemistry, 4–5 months old mice were perfused transcardially with 4% paraformaldehyde (PFA) in PB 0.1 M. Brains were quickly removed, fixed overnight in PFA, and then transferred to 30% sucrose in PBS 0.1 M and stored at 4°C (48 h). Brains were frozen with methylbutane (Honeywell) at −42°C and stored at −80°C until use. Thirty-μm coronal sections were obtained with a freezing microtome (Leica SM2010R) and were kept in a cryoprotective solution at −20°C. Immunohistochemistry was performed on free-floating sections. The sections were inactivated for endogenous peroxidases with 3% H_2_O_2_ in 10% methanol and PBS for 15 min. After three washes with PBS and three washes with PBS-0.2% Triton (PBS-T), sections were blocked for 2 h at room temperature (RT) with PBS-T containing 10% of normal horse serum (NHS) and 0.2% of gelatin. For Reelin immunostaining, anti-mouse unconjugated F(ab′)2 fragments (1:300, Jackson ImmunoResearch), were added in the blocking step. After three washes with PBS-T, tissue sections were incubated with a primary antibody with PBS-T containing 5% of NHS and 0.2% of gelatine, overnight at 4°C.

The commercial primary antibodies used were: anti-Reelin (clone G10, MAB5364, Merck Millipore, 1:1,000), anti-choline acetyltransferase (ChAT AB144P, Merck Millipore, 1:500), anti-μ opioid receptor (MOR, 1:2,000, rabbit, AB5511, Merck Millipore), anti-parvalbumin (PV, 1:500, Rabbit, PV27, Swant), anti-dopamine- and cAMP-regulated phosphoprotein, 32 kDa (DARPP-32, 1:500, mouse, 611520, BD Transduction Laboratories), anti-tyrosine hydroxylase (TH, 1:1,000, Rabbit, AB152, Merck Millipore). Sections were washed with PBS-T and then incubated for 2 h at RT with biotinylated secondary antibody (1:200, Vector Laboratories). After subsequent washes with PBS-T, the sections were incubated for 2 h at RT with streptavidin-HRP (1:400, GE Healthcare UK). After washing, the staining was developed using 0.03% diaminobenzidine (DAB) and 0.01% H_2_O_2_, with 0.1% nickel ammonium sulfate added to the solution. Finally, sections were dehydrated and mounted with Eukitt mounting medium (Sigma-Aldrich).

For immunofluorescence staining a similar procedure was followed using Alexa Fluor 488 secondary antibody (1:500, Invitrogen, ThermoFisher) (excluding peroxidase inactivation), counterstained with Bisbenzimide (1:500) for 30 min at RT, mounted with Mowiol and stored at −20°C.

### 2.5. D1-/D2-cell specific mRNA extraction

Cell-type specific translated-mRNA purification (TRAP), was performed as previously described (Heiman et al., 2008) with a few modifications. Each sample consisted of a pool of 2–3 mice. BAC-TRAP transgenic mice (Drd2- and Drd1a-EGFP) were sacrificed by decapitation. The brain was quickly dissected out and placed in a cold buffer and was then transferred to an ice-cold mouse brain matrix to cut thick slices from which the nucleus accumbens (NAcc) and the dorsal striatum (DS) were punched out using ice-cold stainless-steel cannulas. Each sample was homogenized in 1 ml of lysis buffer (20 mM HEPES KOH (pH 7.4), 5 mM MgCl_2_, 150 mM KCl, 0.5 mM dithiothreitol, 100 μg/ml CHX protease and RNAse inhibitors) with successively loose and tight glass-glass 2-ml Dounce homogenizers. Each homogenate was centrifuged at 2,000 × *g*, at 4°C, for 10 min. The supernatant was separated from cell debris and supplemented with NP-40 (EDM Biosciences) to a final concentration of 1% and DHPC (Avanti Polar lipids) to a final concentration of 30 mM. After mixing and incubating on ice for 5 min, the lysate was centrifuged for 10 min at 20,000 × *g* to separate the supernatant from the insolubilized material. A mixture of streptavidin-coated magnetic beads was incubated with biotinylated protein L and then with GFP antibody that was added to the supernatant and incubated ON at 4°C with gentle end-over rotation. After incubation, beads were collected with a magnetic rack and washed 5 times with high-salt washing buffer (20 mM HEPES-KOH (pH 7.4), 5 mM MgCl_2_, 350 mM KCl, 1% NP-40) and immediately placed in “RTL plus” buffer (Qiagen). The mRNA was purified using the RNase micro KIT (Qiagen). RNA integrity was checked with the Bionalyzer (agilent 2100 Bioanalyzer, Agilent RNA 6000 nano kit). Five nanograms of mRNA from each sample were used for retro-transcription, performed with the Reverse Transcriptase III (Life Technologies) following the manufacturer’s instructions.

### 2.6. Real-time PCR

Quantitative real time PCR, was performed using SYBR Green PCR kit in 96-well plates according to the manufacturer’s instructions. Results are presented as normalized to the indicated house-keeping genes and the delta-threshold cycle (Ct) method was used to obtain a fold change. mRNA levels are presented relative to D2. The housekeeping gene for normalization was beta-myosin heavy chain gene (Myh7).

### 2.7. Western blot

Brains were quickly extracted, frozen in liquid nitrogen and stored at −80°C until use. Brain tissue was processed as previously described (Pujadas et al., 2010). After incubation with antibodies, membranes were developed with the ECL system.

### 2.8. Immunohistochemical analysis

For DARPP-32 cell counting, sections were scanned using NanoZoomer 2.0-HT (Hamamatsu). We used FIJI software to crop the striatal profile from the image. DARPP-32-positive cells were counted with the cell nuclei assistant TMarker software.

The images of PV and ChAT interneurons were acquired with a Nikon E600 microscope attached to an Olympus DP72 camera, and images were reconstructed using MosaicJ from the Fiji software (Fiji is Just ImageJ – NIH). The intermediate striatum was subdivided into four sub-regions: dorso-medial (DM), dorso-lateral (DL), ventro-medial (VM), and ventro-lateral (VL) (see Gernert et al., 2000; Ammassari-Teule et al., 2009) taking slices from Bregma 1.34 to 0.02 mm, to identify possible changes in the neuronal distribution inside the different striatal regions. Cell density studies were performed with FIJI tools to measure the area and to count cells (cell counter).

To measure TH intensity, slides were scanned with SilverFast at 600 ppm and SigmaPlot was used to measure the intensity of the different striatal areas. The results are expressed as % from control which was considered as 1 in each independent experiment to avoid deviations caused by differences in the DAB development procedure.

TH-positive synaptic bouton images were taken with 63X oil immersion objective and counted selecting randomly an 11 mm × 11 mm ROI using Fiji.

For each mouse transgenic line we analyzed 3–14 animals and for each animal and average of 6–8 images were analyzed.

### 2.9. Statistics

All statistical analyses were performed using GraphPad Prism 5.0 software (GraphPad Software, Inc.). Data were analyzed with unpaired, two-tailed Student’s *t*-tests and statistical significance was set at *p*-value < 0.05. Unless otherwise stated, all values are presented as mean ± the standard error of the mean (SEM). The number of animals used in each experiment is detailed in the figure legend.

## 3. Results

### 3.1. Reelin is highly expressed in striatonigral MSNs

**FIGURE 1:**
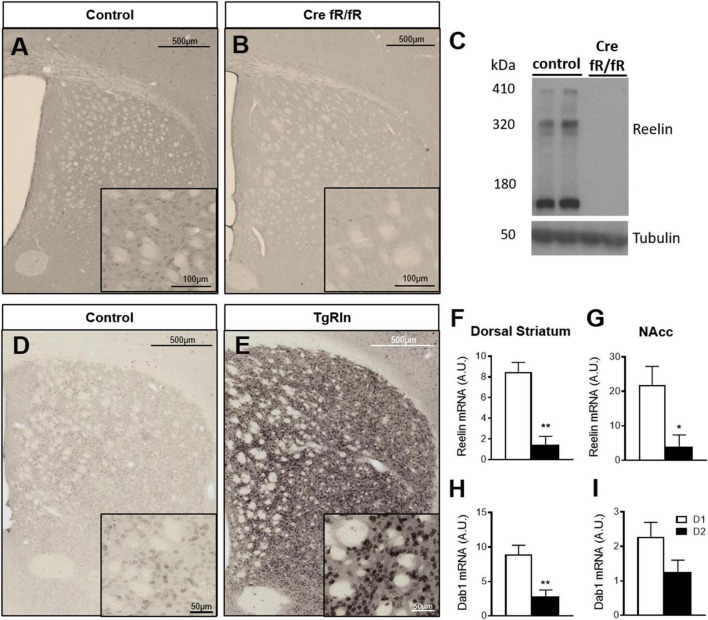
Reelin in the striatum is mainly expressed by D1 striatonigral MSNs. **(A–E)** Immunohistochemistry and Western blot for Reelin show that it is absent in Cre fR/fR mice **(B,C)** compared to the fR/fR littermates (control, **A,C**) while it is clearly overexpressed in the striatum of TgRln mice **(E)** compared to transgenic littermates with an inactive form of the Reelin gene insertion without tTA (controls, **D**). **(F,G)** Quantification of Reelin mRNA levels in the dorsal striatum **(F)** and NAcc **(G)** of D1/D2-TRAP mice (*n* = 3–4). **(H,I)** Quantification of Dab1 mRNA levels in the dorsal striatum **(H)** and NAcc **(I)** of D1/D2-TRAP mice (*n* = 4–7). Scale bar: **(A,B,D,E)**, 500 μm; high magnification insets **(A,B)**, 100 μm, **(D,E)**, 50 μm. NAcc, nucleus accumbens; D1, dopamine 1 receptor; D2, dopamine 2 receptor. Statistical analysis was performed using Student’s *t*-test; significant differences were established at *p < 0.05, **p < 0.01. Data represent means ± SEM.

To determine the effects of Reelin levels in the mouse striatal organization, we first studied Reelin expression in a Reelin overexpressing and a knockout mouse line. Control mice from both lines exhibited numerous Reelin-positive cell bodies that were distributed throughout the striatum (Figures 1A, D), whereas the tamoxifen-inducible conditional knockout mouse line (Cre fR/fR) presented a drastic reduction of Reelin protein as detected by immunohistochemistry (Figure 1B) and by Western blot (Figure 1C). In contrast, Reelin overexpressing mice (TgRln) showed a dramatic increase of Reelin protein in the striatum (Figure 1E) which was apparent in both cell bodies and neuropil (see also Pujadas et al., 2010).

Reelin has been described to co-localize with Calbindin D-28k-positive neurons (Sharaf et al., 2015), a well-known marker of striatal MSNs. Hence, we used the TRAP technology (Heiman et al., 2008) to determine a possible enrichment of Reelin mRNA in D1- or D2-receptor expressing MSNs in both DS and NAcc. BAC-TRAP-D1 and –D2 mice, were used to specifically immunoprecipitate mRNAs from D1 (striatonigral) or D2 (striatopallidal) neuronal populations from the DS and the NAcc. Reelin mRNA levels were compared to the housekeeping beta-myosin heavy chain gene. Results indicated that Reelin mRNA is enriched in D1-MSNs, in both the DS and the NAcc (Figures 1F, G). The expression of Dab1, a key downstream effector of the Reelin pathway, was also higher in D1 MSNs of the DS and NAcc (Figures 1H, I). These results suggest that the striatonigral D1 MSNs population is the main producer of striatal Reelin.

### 3.2. Striatal MSNs organization is independent of Reelin expression levels

**FIGURE 2:**
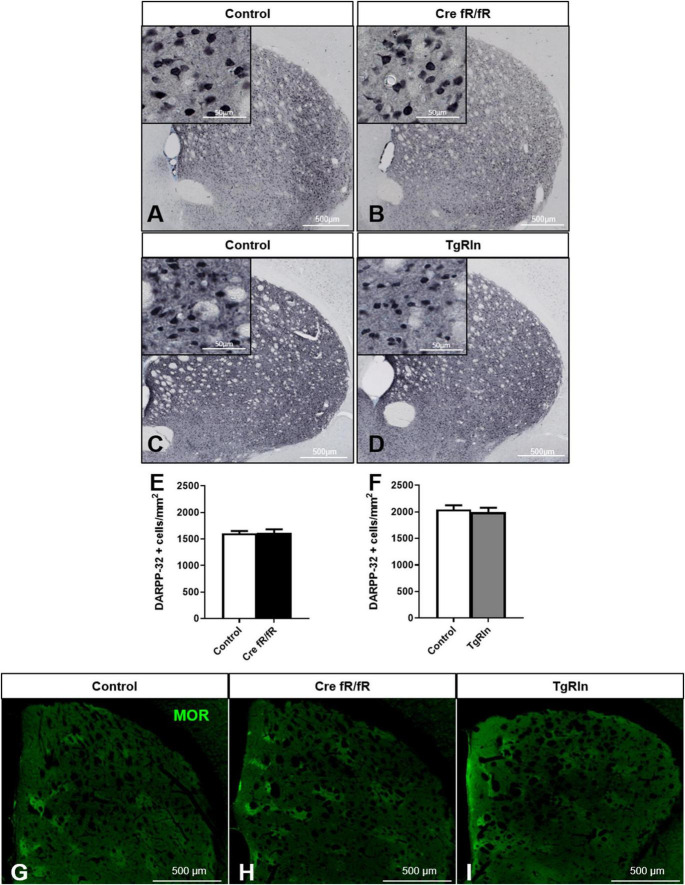
Striatal MSNs density and organization is not affected by Reelin levels. **(A–D)** Representative images of DARPP-32 immunohistochemistry (striatal MSNs) in coronal sections of control and Cre fR/fR **(A,B)** and control and TgRln **(C,D)** striatum (respective controls as in Figure 1). **(E,F)** Quantification of DARPP-32-positive cell density shows no alterations of striatal MSNs neither in Cre fR/fR (*n* = 5–6) **(E)** nor in TgRln (*n* = 6) **(F)** mice. **(G–I)** Immunofluorescence for μ-opioid receptor (MOR) in coronal sections of control **(G)**, Cre fR/fR **(H)**, and TgRln **(I)** striatum showing a similar organization of striatal patches in all the models. Scale bar: **(A–D)**, 500 μm; high magnification insets 50 μm; **(G–I)**, 500 μm. Statistical analysis was performed using Student’s t-test. Data are represented as means ± SEM.

To determine whether Reelin expression levels could modify DS MSN populations, we first immunostained sections with DARPP-32, a marker of MSNs, and quantified the density of striatal MSNs in the Cre fR/fR (Figures 2A, B) and TgRln (Figures 2C, D) mouse models. Results indicated that neither the absence nor the overexpression of Reelin altered the density of striatal DARPP-32 positive neurons in the striatum of Cre fR/fR (Figures 2A, B, E) or TgRln mice (Figures 2C, D, F).

Since Reelin controls neuronal migration, we next wanted to determine whether Reelin levels could affect the DS patch organization. Immunostaining of the striosomes with MOR showed striatal patches with a similar spatial distribution in all genotypes, suggesting that striatal MSNs density and organization are not affected by alterations of Reelin expression levels (Figures 2G–I).

### 3.3. Reelin overexpression alters striatal interneuron population

**FIGURE 3:**
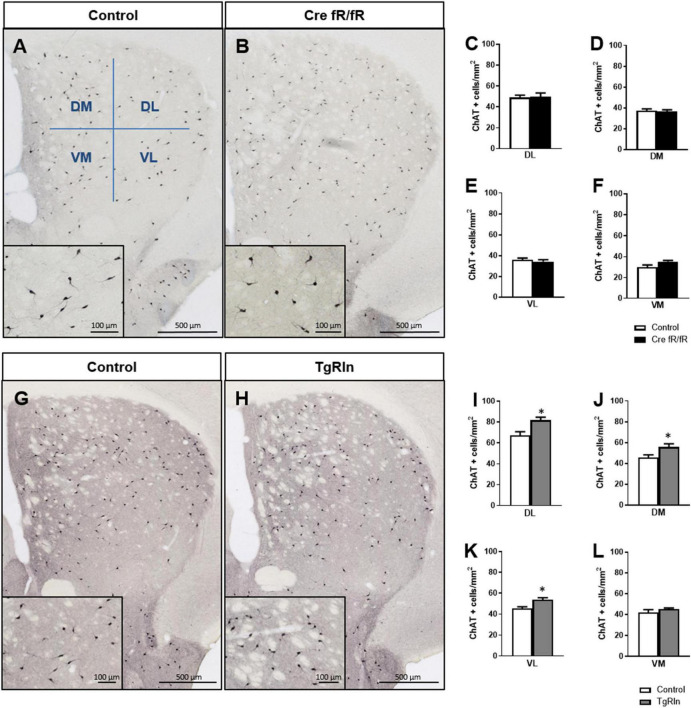
Reelin overexpression increases the density of striatal cholinergic interneurons. **(A,B)** Immunohistochemistry of ChAT in striatal coronal sections of control and Cre fR/fR mice, with representative subdivision of the striatum in four regions (DM, dorsal-medial; DL, dorsal-lateral; VM, ventral-medial; VL, ventral-lateral). **(C–F)** Quantification of ChAT+ neurons density in the striatal subdivisions shows no differences between control and Cre fR/fR mice (*n* = 4). **(G,H)** Representative images of ChAT immunohistochemistry in the striatum of control and TgRln mice, with higher magnification insets showing increased ChAT+ neuronal density in the TgRln mice. **(I–L)** Quantification of ChAT+ cell density indicates a significant increase in the DL, VL, and DM striatal regions of TgRln mice (*n* = 4–6). Scale bar: **(A,B,G,H)**, 500 μm; high magnification insets 100 μm. Statistical analyses were performed using Student’s *t*-test; *p < 0.05. Data are represented as means ± SEM.

In addition to MSNs, the striatum also contains ChAT+ and GABAergic interneurons, the PV-expressing ones being the best known. To assess the number and distribution of ChAT+ interneurons in the different transgenic lines, we subdivided the DS in four sub-regions: DM, DL, VM, and VL (Figure 3A). Analysis of the density and distribution of ChAT+ cells showed no differences in Cre fR/fR mice compared to controls (Figures 3A–F). In contrast, the density of ChAT+ cells was increased in Reelin overexpressing mice compared to controls, reaching significance in 3 of the striatal sub-regions analyzed (Figures 3G–L).

**FIGURE 4:**
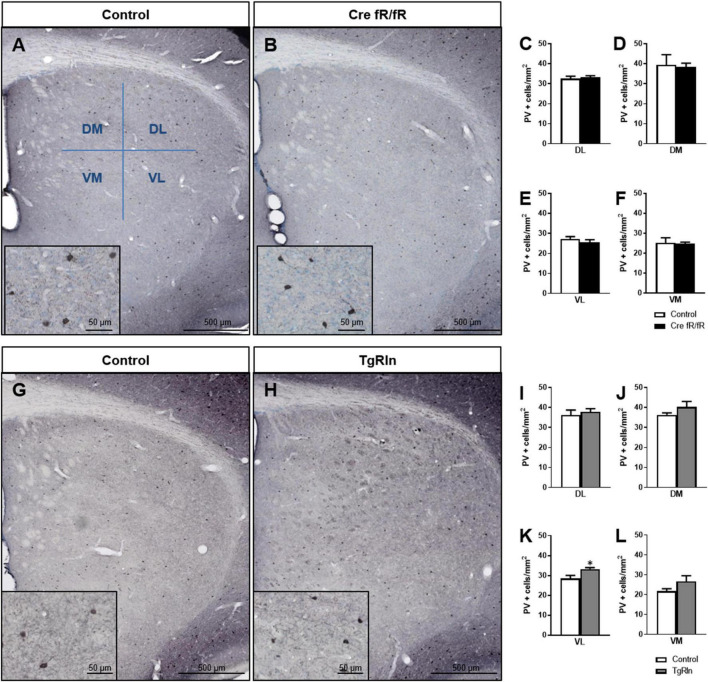
Increased levels of Reelin alter the density of parvalbumin interneurons in the ventral-medial striatum. **(A,B)** Immunohistochemistry for PV in coronal sections of control and Cre fR/fR striatum, subdividing the striatum in four regions (as in Figure 3). **(C–F)** Quantification of the density of PV+ interneurons indicated no differences between the control and Cre fR/fR mice (*n* = 4). **(G,H)** Representative images of PV immunostaining in the striatum of control and TgRln mice. Quantification of PV immunohistochemistry indicated an increase in the density of PV positive cells in the VL striatum of TgRln mice (*n* = 4–5) **(K)** with no differences in the other striatal regions **(I,J,L)**. Scale bar: **(A,B,G,H)**, 500 μm; high magnification insets 50 μm. Statistical analyses were performed using Student’s *t*-test; *p < 0.05. Data are represented as means ± SEM.

We also analyzed the density and distribution of PV striatal interneurons. In line with the ChAT+ interneuron data, no changes in the density and distribution of PV+ interneurons (Figures 4A, B) were observed in any of the DS regions of Cre fR/fR mice compared to controls (Figures 4C–F). However, analysis of PV+ interneuron density in TgRln mice showed a statistically significant increase in the VL striatum (Figures 4G, H, K) but not in other striatal regions (Figures 4G–J, L) as compared to controls. Altogether, our results indicated that Reelin overexpression increased the number of DS interneurons.

### 3.4. Reelin levels control dopaminergic projections

**FIGURE 5:**
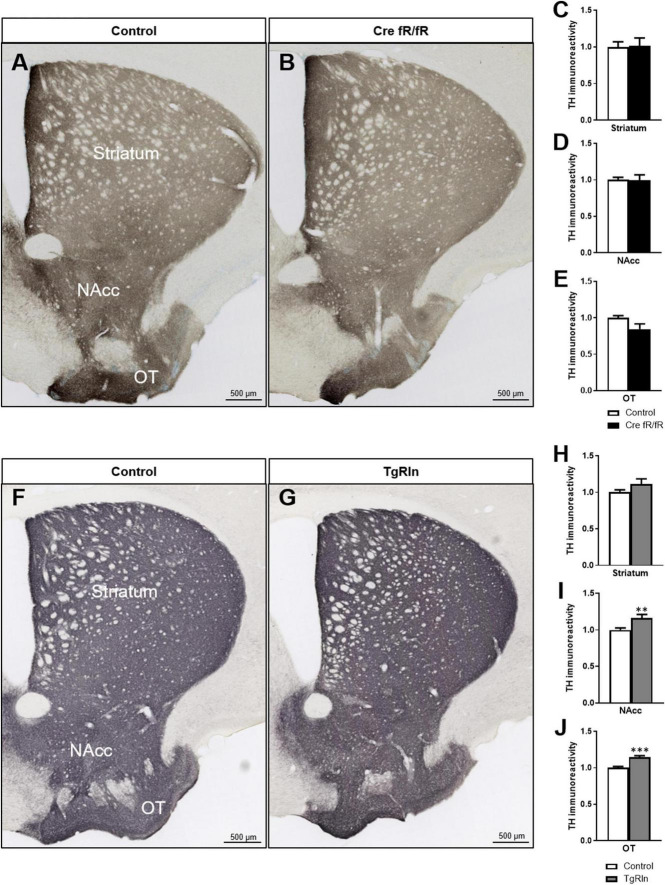
Increased levels of Reelin elevates dopaminergic projections in the ventral striatum. **(A,B)** Immunohistochemistry for TH to stain dopaminergic projections in coronal sections of the DS, NAcc, and OT of control and Cre fR/fR mice. TH intensity remains constant in the striatum **(C)**, NAcc **(D)**, and OT **(E)** of Cre fR/fR mice compared to controls (*n* = 4). **(F,G)** Immunohistochemistry for TH in control and TgRln mice. After quantification, increased TH immunoreactivity was detected in the NAcc **(I)** and OT **(J)**, but not in the DS **(H)** of TgRln mice compared to controls (*n* = 8–14). Scale bar: **(A,B,F,G)**, 500 μm. NAcc, nucleus accumbens; OT, olfactory tubercle. Statistical analyses were performed using Student’s *t*-test; **p < 0.01; ***p < 0.001. Results represent the means ± SEM.

Next, we analyzed whether the expression of Reelin could influence dopaminergic projections. Thus, we performed immunohistochemistry for TH to detect dopaminergic projections that reach the striatum from the SN and the ventral tegmental area (VTA). We quantified TH intensity in the DS and the ventral striatum (VS), including the NAcc and the olfactory tubercle (OT). In the Cre fR/fR model, we observed no alterations in the dopaminergic intensity in all three striatal regions studied (Figures 5A–E) compared to controls. However, in the OT of Cre fR/fR mice, we observed a tendency toward a reduction in TH intensity compared to controls (Figure 5E). In contrast, in Reelin overexpressing mice, quantification of TH immunostaining (Figures 5F, G) showed a significant increase of TH intensity in both the NAcc and OT regions compared to controls (Figures 5H–J).

**FIGURE 6:**
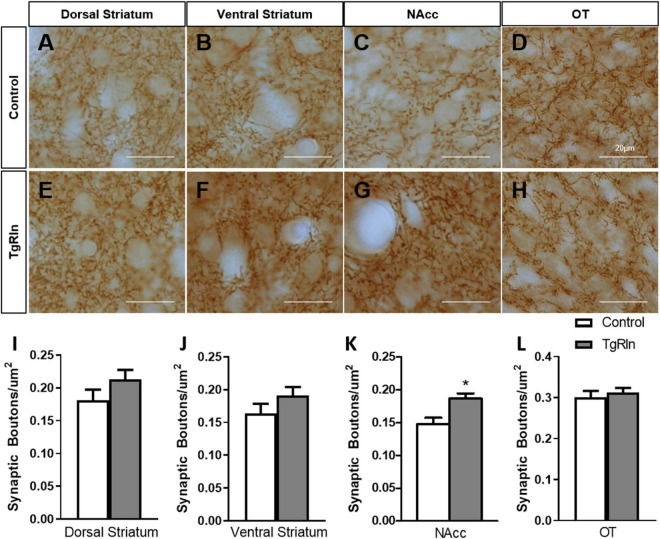
Increased number of dopaminergic synaptic boutons in the NAcc of TgRln mice. **(A–H)** Immunohistochemistry for TH staining dopaminergic synaptic boutons in the dorsal **(A,E)** and ventral regions **(B,F)** of the DS, NAcc **(C,G)**, and OT **(D,H)** of TgRln mice and its controls. **(I–L)** Quantification of the density of dopaminergic boutons evidenced a higher density of synaptic boutons in the NAcc **(K)** of TgRln mice compared to its controls while no differences were observed in the rest of the analyzed structures **(I,J,L)**. Scale bar: **(A–H)**, 20 μm. DS, dorsal striatum; NAcc, nucleus accumbens; OT, olfactory tubercle. Statistical analyses were performed using Student’s *t*-test; *p < 0.05. Data are represented as means ± SEM.

Finally, we also wanted to quantify synaptic boutons of striatal dopaminergic projections. Thus, we determined the density of TH-positive synaptic boutons in the DS, NAcc, and OT, dividing the DS into dorsal and ventral regions. In the Cre fR/fR mice, the density of synaptic boutons in all the regions was similar to that of control mice (DS dorsal: fR/fR 0.28 ± 0.017 vs. Cre fR/fR 0.30 ± 0.014; DS ventral: fR/fR 0.27 ± 0.016 vs. Cre fR/fR 0.27 ± 0.023; NAcc: fR/fR 0.26 ± 0.013 vs. Cre fR/fR 0.25 ± 0.034; OT: fR/fR 0.24 ± 0.013 vs. Cre fR/fR 0.25 ± 0.018; *n* = 4 mice/genotype, mean ± SD). In contrast, the density of dopaminergic synaptic boutons were all increased in the tested regions (Figures 6A–L), but only the NAcc showed a statistically significant increase (Figures 6C, G, K) in the TgRln mice as compared to controls. These results suggest that higher Reelin levels might modulate dopaminergic fibers and synaptic boutons, mainly in the NAcc.

## 4. Discussion

**FIGURE 7:**
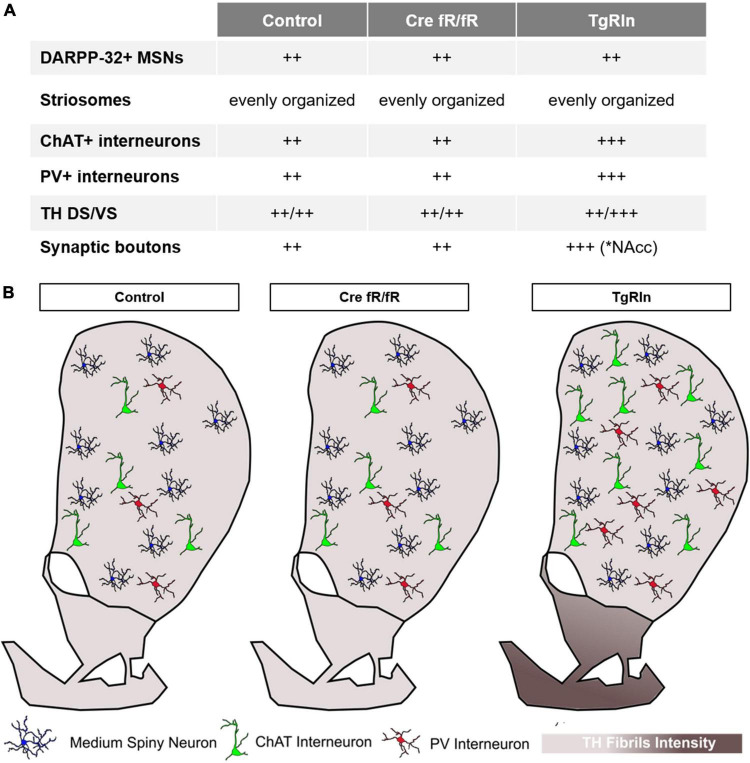
Schematic summary of the striatal organization in different Reelin mouse models. **(A)** Summary table indicating the main changes observed in striatal organization in the different strains. **(B)** Graphic summary of the striatal organization per strain. Density of striatal MSNs is preserved between the control, Cre fR/fR, and TgRln striatums. Although the density of striatal PV+ and ChAT+ interneurons is maintained between control and Cre fR/fR mice, it is increased in the DS of TgRln mice. Increased numbers of ChAT+ interneurons are present in the dorsal striatum and higher numbers of PV+ interneurons are distributed in the ventral medial striatum sub-region. Dopaminergic projections are represented with different gradient of brown color, showing a specific increase of TH fibers in the NAcc and OT of the TgRln mice compared with controls.

Variations in Reelin expression levels have been shown to be important for the development of neuropsychiatric disorders (Impagnatiello et al., 1998; Fatemi et al., 2000, 2001, 2005; Persico et al., 2001; Grayson et al., 2005; Torrey et al., 2005; Ruzicka et al., 2007; Ovadia and Shifman, 2011; Wang et al., 2014; Baek et al., 2015; Lammert and Howell, 2016); however, we still lack a precise understanding of the mechanistic insights of this correlation. Here we focused our attention on the striatum as a key region participating in the pathogenesis of psychiatric diseases (McCutcheon et al., 2021). We thus characterized specific striatal neuronal populations as well as the dopaminergic mesolimbic innervation in two different mouse models either overexpressing or deficient for Reelin. In previous studies we reported that TgRln mice were more resilient to stressors implicated in the genesis of psychiatric diseases (chronic stress and psychostimulant administration) (Teixeira et al., 2011), suggesting a role for Reelin in preventing behavioral symptoms related with these disorders. Here we show that Reelin-depletion at adult stages does not lead to significant changes either in the striatal composition or in dopaminergic innervation, suggesting that during adulthood Reelin is not essential for the maintenance of striatal organization. However, postnatal Reelin overexpression increases interneuron populations as well as the density of dopaminergic striatal projections from the VTA, suggesting the participation of postnatal Reelin expression in the fine structural tuning of the striatal area (Figure 7).

### 4.1. A role for Reelin in the striatum

The role of Reelin in the cortex and the hippocampus has been extensively studied including the expression pattern in GABAergic interneurons and the regulation in glutamatergic synapses (Alcántara et al., 1998; Herz and Chen, 2006; Jossin, 2020). Indeed, it has been shown that Reelin controls several structural and functional properties of the glutamatergic synapses including the strength of glutamate neurotransmission (Beffert et al., 2005; Qiu et al., 2006b), protein composition of presynaptic boutons (Hellwig et al., 2011), structural properties of dendritic spines (Bosch et al., 2016) as well as trafficking of glutamate receptor subunits (Sinagra et al., 2005; Groc et al., 2007). Several studies also support a key role of Reelin in the correct organization of the basal ganglia. Blockade of Reelin or its signaling pathway leads to a severe disorganization of the tangentially migrating midbrain dopaminergic (mDA) neurons, which fail to reach their final position in the SN pars compacta (SNc) and accumulate instead in the VTA, resulting in a conspicuous reduction of mDA neurons in the SNc, despite no overall changes in the number of mDA neurons (Nishikawa et al., 2003; Kang et al., 2010; Sharaf et al., 2013; Bodea et al., 2014). Interestingly, alterations in the radial and tangential fibers that guide migrating mDA neurons have been described in *reeler* mice (Nishikawa et al., 2003; Kang et al., 2010) and specific inactivation of Reelin signaling in mDA neurons indicates a direct role of Reelin by promoting fast-laterally directed migration and stabilization of their leading process (Vaswani et al., 2019). Despite these organization abnormalities in the SNc, no significant alterations have been described in the nigrostriatal pathway of *reeler*, *reeler-like* mutants or heterozygous *reeler* mice (Nishikawa et al., 2003; Sharaf et al., 2013; Vaswani et al., 2019). In contrast, defects in cortico-striatal plasticity (Marrone et al., 2006) and in the dopaminergic system (Matsuzaki et al., 2007) have been reported in *reeler* mice. Moreover, decreased levels of Reelin have been associated with alterations in striatal composition, such as reductions in the number of striatal PV+ neurons along the rostro-caudal axis (Marrone et al., 2006; Ammassari-Teule et al., 2009), decreases in TH immunoreactivity in the striatum, VTA, and NAcc (Ballmaier et al., 2002; Nullmeier et al., 2014) and increases in the density of ChAT (Sigala et al., 2007) and the expression of D1, D2, and serotonin 5-HT2A receptors (Matsuzaki et al., 2007; Varela et al., 2015). Importantly, it is still controversial whether these striatal alterations are attributable either to a role of Reelin during development or to an acute effect at adult stages. Given that very few studies have addressed this issue (Matsuzaki et al., 2007), here we use a conditional KO model (Cre fR/fR) in which neurodevelopment is preserved, which allowed us to specifically analyze the contribution of adult Reelin expression to the cellular and anatomical organization of the striatum. Our results indicate no significant changes either in the cell densities of CHAT+ and PV+ interneurons or in the density of DARPP32+ MSNs, suggesting that Reelin expression is critical for striatal composition during development, but not for the maintenance of cellular pools during adulthood. Moreover, mapping of the TH+ immunoreactivity in DS, VTA, and NAcc areas in Cre fR/fR mice showed no differences from controls, although there was a trend in the OT, supporting that at adult stages Reelin is largely dispensable for the maintenance of the dopaminergic innervation from the SN/VTA to the striatum.

### 4.2. Reelin, psychiatric behavioral phenotypes, and drug sensitization

Alterations in Reelin levels have been associated with psychiatric-related behavioral phenotypes in rodents, including visual attention-related deficits in reversal learning (Brigman et al., 2006), decreased inhibition, emotionality, and motor impulsivity starting from adolescence (Ognibene et al., 2007), sensorimotor gating impairments (Barr et al., 2008) or altered latent inhibition (Ammassari-Teule et al., 2009), although these results are controversial since many labs have failed to reproduce them (Podhorna and Didriksen, 2004; Teixeira et al., 2011; Lossi et al., 2019). Interestingly, pharmacological supplementation or genetic overexpression of Reelin, prevent some of these deficits (e.g., associative learning, sensorimotor gating) (Teixeira et al., 2011; Rogers et al., 2013) and support the idea of boosting Reelin levels as a therapeutic approach in psychiatric disorders. Moreover, several studies relate Reelin deficits with exacerbated behavioral abnormalities in response to drug consumption (Romano et al., 2014; Iemolo et al., 2021; de Guglielmo et al., 2022) and we have previously reported that Reelin overexpression prevents psychomotor effects associated with chronic psychostimulant administration (Teixeira et al., 2011), supporting a role for Reelin in drug sensitization. Importantly, there is a high comorbidity between substance use disorders (cannabis, nicotine, alcohol, and stimulants) and schizophrenia, with an estimated prevalence of 41.7% (Hunt et al., 2018).

It has been widely described that the mesolimbic system is the major neurochemical pathway controlling the rewarding effects of drugs of abuse (Wise and Rompre, 1989). Disturbances in the dopaminergic mesolimbic system including altered immunoreactivity and mRNA levels of TH and dopamine receptors (D2 and D3) in the VTA and the VS have been reported in heterozygous *reeler* mice (Ballmaier et al., 2002) and could be related to some of the behavioral deficits observed in this model. In this regard, our results show an increased immunoreactivity of dopaminergic fibers in the NAcc and OT of the TgRln mice which could be indicative of increased dopamine (DA) release from the VTA. The NAcc, is one of the main projection sites of the VTA, and the release of DA in this region is associated with reward and motivated behaviors (Robison et al., 2020). The OT, on the other hand, is markedly connected with sensory and arousal/reward centers and is critical for multisensory integration and hedonic responses (Wesson and Wilson, 2011). Human imaging studies, pharmacology and preclinical models indicate that the integration of the reward circuitry is affected in schizophrenia (Robison et al., 2020) and a higher reactivity of dopamine release appears to play a role in schizophrenia positive symptoms through its action on D2 receptors, including the NAcc (see Simpson et al., 2022 for a review). Importantly, there are studies involving other striatal elements, such as the striatal patch-matrix organization and striatal interneurons, in the control of reward and drug abuse, making the characterization of the striatal organization in TgRln mice essential to further understand the mechanisms underlying drug sensitization. Despite the fact that the gross structure of the striatal architecture was not altered in TgRln mice, the study of striatal interneurons, which represent 5–10% of the striatal cell population, clearly suggests that Reelin is able to modulate interneuron densities. Reelin overexpression leads to increased densities of PV+ and ChAT+ cells, suggesting a specific response of these neurons to increased amounts of Reelin. Noteworthy, schizophrenic patients present reduced densities of ChAT+ interneurons in the caudate nucleus, the VS and in the striatum as a whole (Holt et al., 1999, 2005).

Decreased density of PV+ interneurons in the dorsomedial and ventromedial striatum of heterozygous *reeler* mice have been paralleled with deficits in some behaviors strongly disrupted in schizophrenic patients (Ammassari-Teule et al., 2009). Moreover, cocaine sensitization correlates with transient increases in the number of PV+ neurons in striatum, which is reduced below the normal number after a 2-week cocaine withdrawal period (Todtenkopf et al., 2004). The fact that TgRln mice, which show reduced sensitization to cocaine, also show increased densities of PV+ interneurons could appear contradictory; nevertheless, here the increased number of PV+ interneurons is sustained, while upon cocaine administration the increase is transient, and eventually, related to compensatory responses. In addition, recent data suggest that increased acetylcholine signaling reduces the acute and sensitized motor responses to cocaine (Lewis et al., 2020) supporting the idea that the increased PV+ and ChAT+ interneuron density observed in TgRln mice could be involved in the reduction of cocaine sensitization described in these mice (Teixeira et al., 2011).

It has been described that after cocaine administration, there is an specific increase in the ERK pathway in striatonigral MSNs (Bertran-Gonzalez et al., 2008), a pathway that is also activated by Reelin (Simó et al., 2007; Lee et al., 2014). Interestingly, an increased Fos activation in the dorsal medial striatum but not in the NAcc of heterozygous *reeler* mice after the administration of cocaine has been reported (de Guglielmo et al., 2022). Increases in Fos activation are thought to be the result of the cocaine-induced upregulation in dopamine levels in the striatum (Di Chiara and Imperato, 1988), which is hypothesized to alter the activity of MSNs by activating D1 and D2 receptors. Experiments in mice lacking D1 receptor indicate a clear role for this receptor in the psychomotor effects of cocaine. In this study we describe a preferential expression of Reelin mRNA in a specific subpopulation of MSNs of the striatum, the D1 neurons, corroborating previous studies using FISH (de Guglielmo et al., 2022) or genome-wide translatome (Montalban et al., 2022). The fact that the expression of both Reelin and its main downstream effector Dab1 are higher in striatonigral D1 MSNs than in striatopallidal D2 MSNs, suggests that Reelin may function in an autocrine manner in D1 MSNs and could be somehow modulating its function and hence influencing cocaine-induced psychomotor effects which are reduced in Reelin overexpressing mice (Teixeira et al., 2011) and increased when Reelin levels are reduced (de Guglielmo et al., 2022).

Although the mechanisms by which Reelin overexpression leads to increased numbers of PV and CHAT neurons remain unknown, it is important to remark that CAMKII promoter drives expression of Reelin in the striatum from the end of the first postnatal week onward (P5–P10) (Pujadas et al., 2010). Hence, we discard direct actions of Reelin overexpression on neurogenesis and migration, since those processes take place mainly at embryonic stages (Knowles et al., 2021). Several recent studies using conditional Reelin inactivation either from postnatal stages or specifically targeting interneurons, have indicated that Reelin participates in the fine tuning of cortical and hippocampal layering (Pahle et al., 2020; Vílchez-Acosta et al., 2022) and in dendritic growth control of specific interneuronal subsets through the regulation of presynaptic neurotransmitter release and Ca^2+^ influx (Hamad et al., 2021). It has been recently reported that PV+ and ChAT+ striatal interneurons undergo extensive apoptosis during the first and second postnatal weeks, with the main changes occurring between P5 and P10 (Sreenivasan et al., 2022), coinciding with the onset of Reelin overexpression in our model. The survival of these particular interneuronal populations has been related with their specific afferent connectivity, being PV+ interneurons controlled by long-range cortical inputs whilst local inputs from MSNs control ChAT+ interneurons. Our data indicate that Reelin overexpression does not influence the total number of DARPP-32+ MSNs, however, as described in other brain regions such as the hippocampus (Pujadas et al., 2010), Reelin overexpression could be modulating neuronal activity levels and hence affecting interneuron survival. Moreover, it is also possible that Reelin influences positively the maturation and survival of these interneurons, through Reelin/Dab1 associated pathways that are known to influence these processes (Simó et al., 2007; Lee et al., 2014). Nevertheless, since PV and ChAT expressions can be modulated by many factors, we cannot completely exclude the possibility that Reelin overexpression might be increasing PV and ChAT expression and therefore making visible interneurons that otherwise express very low marker levels in control mice.

### 4.3. Reelin as a possible therapeutic target for psychiatric diseases

Reelin has been placed as a top candidate gene associated with several neuropsychiatric diseases. This link is supported by several studies showing that Reelin levels are reduced in patients with schizophrenia, bipolar disorder, and autism spectrum disorder (Impagnatiello et al., 1998; Fatemi et al., 2000, 2001, 2005; Persico et al., 2001; Grayson et al., 2005; Torrey et al., 2005; Ruzicka et al., 2007; Ovadia and Shifman, 2011; Wang et al., 2014; Baek et al., 2015; Lammert and Howell, 2016). Excitatory/inhibitory unbalances have been widely reported in schizophrenia patients and mouse models (Gao and Penzes, 2015). Current data indicates decreased GABA and increased glutamate levels in schizophrenic patients (Rowland et al., 2013; Song et al., 2014; Chiu et al., 2018) that most likely lead to hyperexcitability in certain brain circuits. Interestingly, the increased density in GABAergic PV+ and ChAT+ interneurons observed in TgRln mice presumably intensifies the inhibitory input onto striatal MSNs, reducing in turn the excitability in the striatal circuitry and perhaps counteracting in part the excitatory/inhibitory imbalance typically found in schizophrenia.

It is interesting to note that the striatal changes observed in TgRln mice are opposite to those found in patients with Tourette’s syndrome which present a clear decrease in the density of PV+ and ChAT+ interneurons in the DS with no alterations in the density and number of MSNs (Kataoka et al., 2010). The fact that GWAS studies have identified RELN genetic variants in Tourette’s syndrome (Li et al., 2012) together with our findings in the TgRln model suggest that Reelin overexpression could reverse some of the symptoms of this disorder, although altered Reelin expression or signaling should be explored in patients affected by Tourette’s syndrome.

## Data availability statement

The original contributions presented in this study are included in the article/supplementary material, further inquiries can be directed to the corresponding authors.

## Ethics statement

This animal study was reviewed and approved by the University of Barcelona Animal Ethics Committee and the Rockefeller University’s Institutional Animal Care and Use Committee.

## Author contributions

ES contributed to conception and design of the study. MP, SG, EM, LP, AE-T, NM, AV-A, and YM performed the experiments. MP, SG, EM, CA, and YM analyzed the data and performed the statistical analysis. AN contributed to new reagents/analytic tools. MP, LP, YM, and ES wrote the first draft of the manuscript. EM, AP, J-AG, CA, and MV wrote sections of the manuscript. YM and ES reviewed and edited the final version. All authors contributed to manuscript revision, read, and approved the submitted version.
